# Advancement in Dietary Assessment and Self-Monitoring Using Technology

**DOI:** 10.3390/nu11071648

**Published:** 2019-07-19

**Authors:** Tracy L. Burrows, Megan E. Rollo

**Affiliations:** 1 School Health Science, Faculty of Health and Medicine, University of Newcastle, Callaghan, NSW 2308, Australia; 2 Priority Research Centre for Physical Activity and Nutrition, University of Newcastle, Callaghan, NSW 2308, Australia

## 1. Introduction

On the surface, some methods to assess and self-monitor dietary intake may be considered similar; however, the intended function of each is quite distinct. Methods used in the assessment of dietary intake aim to measure food and nutrient intake and/or derive dietary patterns for determining diet-disease relationships, conduct population surveillance, or determine the effectiveness of interventions [1]. In comparison, dietary self-monitoring primarily aims to create awareness and reinforcement of individual eating behaviours, in addition to tracking foods consumed, and has been particularly useful in the context of weight management [2]. Advancements in the capabilities of technologies, such as smartphones and wearable devices, have enhanced the proficiencies of collection, analysis, and interpretation of dietary intake data in both contexts across the spectrum of users, including consumers, clinicians and researchers.

In this issue, a range of new articles are presented, and we are fortunate to have a collection of reviews and empirical studies to assist in the development of understanding and attainment of new knowledge to assist in progressing this area of research on the use of technology in dietary assessment methods.

This special issue includes five review papers. Two articles reviewed mobile/smartphone applications [3,4], including the potential of mHealth apps to increase fruit and vegetable intake [3]. This specific review included eight studies, six of which were effective in increasing fruit and/or vegetable intake [3]. Additionally, a second paper included a review of recipe functions in 12 popular dietary smartphone apps and found a large variation in their energy and macronutrient calculations [4]. The main variation between apps occurred at the analysis phase due to the type of food composition table used to generate nutrient values [4].

A narrative review of new methods for assessing food and energy intake [5] is presented along with a review on the evaluation of new technology-based tools for dietary intake assessment [6]. This review of technology-based diet assessment tools, which included tools categorised for both research and consumer use, showed that the majority (79%) relied on self-reported dietary intakes. Most (91%) used text entry, 33% used image-based methods, 65% had integrated databases to estimate energy or nutrients, and less than 50% had customisation features [6]. Technology-based dietary assessment offers many advantages for research, and is often preferable to consumers over more traditional methods.

In addition, a narrative review in this special issue presents a synthesis of data on the dietary assessment of shared plate eating, which is reported as a missing link within a large proportion of methods that collect or focus on individual intake only [7]. Shared plate eating is reported as a particular issue for low-and lower-middle income countries where this type of eating behavior is common. Most studies used 24-h recalls—many used tools to assist in quantifying food intake, including food photographs and images of portion sizes [7]. The gap in this area of research was identified, as well as practical set of recommendations provided to move the field forward.

Finally, a systematic review on upper limb sensors for the assessment of eating behaviour summarises the findings from 69 studies [8]. To date, the majority of studies in the area have been conducted in controlled environments, among young, healthy individuals, and using accelerometers in combination with gyroscopes to detect eating activity. Heydarian and colleagues suggest the development of large datasets are paramount to advancing the field, particularly with regard to the use of deep learning for the detection of different eating gestures.

The empirical studies in this special issue can be classified into a number of key areas and are summarised below:

## 2. Web-Based Dietary Assessment Methods

Three articles are presented around the use of web-based methods, which in this special issue were applied in two of three studies to the 24-h recall method [9,10,11]. Interestingly, two studies explored the useability of technology-based methods in populations where use and acceptability might be questioned [9,10]. In one study by Polfuss et al., the usability of a technology-based 24-h recall was explored in individuals with and without disabilities, showing the methods were acceptable [10]. Another study in low income adults identified a range of useability issues with the automated self-administered dietary assessment tool (ASA24), including the misunderstanding of questions and uncertainties concerning how to proceed to the next step [9]. These papers provide very practical suggestions when applying technology to dietary assessment methods in these populations.

## 3. Image-Based or Image-Assisted Methods

Image-based or image-assisted methods were used to capture dietary intake and are were reported in this special issue in three studies [12,13,14], two of which were carried out in pediatric populations [13,14]. A validation study in young infants investigated the accuracy of image-assisted food records versus regular food records compared to the objective marker of doubly labelled water (DLW) method [13]. Another study in children of primary school age (9–12 years) investigated the accuracy of an electronic image-based food diary compared with a paper-based food diary over a four-day collection period [14]. The image-based food diary used a combination of photographs and written descriptions of foods consumed. Similar results were found for macro-and-micro nutrients for both methods. However, the image-based food diary was less burdensome for researchers and participants—it was also preferred by the children, and they required less help completing it [14].

## 4. Mobile/Smartphone Applications for Capturing Intake or Self-Monitoring

An interesting collection of articles are presented which range from quantitative, qualitative, and mixed method evaluations of the use of applications for dietary self-monitoring. For quantitative evaluation, the relative validity of the eat and track (EaT) smartphone app for the collection of dietary intake data was explored in young adults aged 18 to 30 years [15]. This population group is often difficult to engage in dietary and lifestyle interventions despite their known weight gain trajectory to be higher than any other population group. In this group the app was compared with dietitian-administered 24-h recalls. Significant differences in dietary energy were found but an agreement for most nutrient densities were reported at the group level. In another study, the effectiveness of the nutritional app “MyNutriCart” was reported and compared to a traditional face-to-face counselling session in order to determine the differences in food choices related to purchase and dietary behavior [16]. While in this pilot study there were no differences between groups, “MyNutriCart” did lead to significant improvements in household purchasing behaviours and individual intakes compared to baseline [16].

The Bridge2U mobile app food log was compared to control meal and dietary recall methods in another study [17]. While carried out in small population group (*n* = 14), the Bridge2U was reported as a good dietary assessment method for the assessment of intake at the group level, but data was reported to be highly variable for individual assessment [17]. Qualitative data provides very useful insights that often cannot be obtained through quantitative measurements only. An interesting study in this special issue reports on a qualitative evaluation of the eaTracker^®^ Mobile App [18]. Structured interviews of 26 participants were analysed to evaluate ways to improve the eaTracker and provide information for those looking to develop apps to facilitate positive behaviour change. A number of positive aspects, challenges and suggestions for improvement of the app were collected and reported [18]. An evaluation of mixed methods is reported in a study by den Braber et al. to comprehensively determine the requirements of “The Diameter”: an app to monitor diet, physical activity, and glucose values in patients with Type 2 Diabetes [19]. The study provides useful insight for this population group.

Mobile apps can be used to collect images, but they can also be used to collect voice recordings. Voice recordings can be used to add details to images which may often not be apparent and/or be used to collect information where an image of a food or drink maybe missed. An interesting study describes a voice operated app to determine the accuracy of automatic carbohydrate, protein, fat, and calorie counting based on the voice descriptions of meals in people with Type 1 Diabetes [20]. In 30 patients, insulin doses were estimated by a physician using dietary data obtained from VoiceDiab (*n* = 16) and this was compared to dietary data provided by a dietitian (*n* = 14). No significant differences in insulin doses or glycaemic control were reported using either system [20].

Wearable cameras are considered a passive technology as opposed to active capture whereby an individual still needs to be actively involved in the process. Passive measures can reduce the burden for participants in collecting dietary intake data, however, researcher burden still exists in other stages of dietary assessment, such as image processing and quantification. In one study of this special issue, a wearable system called the automatic ingestion monitor (AIM) was used to detect and monitor participant food intake (*n* = 40) for three days [21]. This was validated by a comparison with video observation that was annotated by three researchers to report activities, resting, walking, chewing, and biting during each eating and drinking episode [21].

If we look at the technology being applied to the analysis part of dietary assessment rather than the collection phase, when many other papers report on this in this special issue, one study compared the nutrient estimates based on food volume versus weight [22]. The weights of 35 individual food volumes were measured (control) and compared to the USDA-SR weights. Significant differences were found for 80% of foods which suggests that USDA-SR may not provide accurate estimates of dietary intake when assessed using food volumes [22].

This special issue presents a great set of articles regarding technology-based issues in the collection, analysis, and interpretation of dietary data.
